# Molecular Dynamics Simulation of Distribution and Diffusion Behaviour of Oil–Water Interfaces

**DOI:** 10.3390/molecules24101905

**Published:** 2019-05-17

**Authors:** Chengbin Zhang, Hanhui Dai, Pengfei Lu, Liangyu Wu, Bo Zhou, Cheng Yu

**Affiliations:** 1 Key Laboratory of Energy Thermal Conversion and Control of Ministry of Education, School of Energy and Environment, Southeast University, Nanjing 210096, China; cbzhang@seu.edu.cn (C.Z.); hhdai@microflows.net (H.D.); 2 School of Hydraulic, Energy and Power Engineering, Yangzhou University, Yangzhou 225127, China; pengfeilu@dicp.ac.cn (P.L.); lywu@yzu.edu.cn (L.W.); 3 Dalian National Laboratory for Clean Energy, Dalian Institute of Chemical Physics, Chinese Academy of Sciences, Dalian 116023, China; 4 Jiangsu Key Laboratory of Micro and Nano Heat Fluid Flow Technology and Energy Application, School of Environmental Science and Engineering, Suzhou University of Science and Technology, Suzhou 215009, China; zhou-b12@tsinghua.org.cn

**Keywords:** concentration distributions, interface roughness, oil–water interface, diffusion

## Abstract

The distribution and diffusion behaviors of microscopic particles at fluorobenzene–water and pentanol–water interfaces are investigated using molecular dynamics simulation. The influences of Na^+^/Cl^−^ ions and the steric effects of organic molecules are examined. The concentration distributions of different species, the orientations of oil molecules at the interface, and oil–water interface morphology as well as the diffusion behaviors of water molecules are explored and analyzed. The results indicate that a few fluorobenzene molecules move into the water phase influenced by Na^+^/Cl^−^ ions, while the pentanol molecules at the interface prefer orientating their hydrophilic groups toward the water phase due to their large size. The water molecules more easily burst into the pentanol phase with larger molecular spaces. As the concentration of ions in the water phase increases, more water molecules enter into the pentanol molecules, leading to larger interface roughness and interface thickness. In addition, a lower diffusion coefficient for water molecules at the fluorobenzene–water interface are observed when introducing Na^+^/Cl^−^ ions in the water phase, while for the pentanol–water system, the mobility of interfacial water molecules are enhanced with less ions and inhibited with more ions.

## 1. Introduction

Oil–water interfaces exist widely in physical, chemical, and biological processes, such as interface adsorption [1], extraction [2], emulsification [3,4], phase transfer [5,6], and component transport [7,8]. From a microscopic point of view, the particle diffusion at the oil–water interface is simultaneously affected by the same and heterogeneous phases nearby in the interface, thus presenting distribution and diffusion characteristics that differ from the particles inside the substance. This directly affects the micromorphology of the oil–water interface and the diffusion efficiency at the interface. Therefore, the investigation of the distribution and diffusion of microscopic particles at the oil–water interface is not only of scientific significance for understanding the structure and physicochemical characteristics of the oil–water interface, but also to provide key technical support for the regulation and optimization of various interface processes in practical applications [9,10,11,12].

Since the 19th century, extensive experimental studies have been conducted on interfacial tension, solution adsorption, liquid membrane separation, and other interfacial properties and processes [13,14]. The rapid development of modern surface/interface analysis techniques (such as surface microscopy, surface spectroscopy, etc.) has pushed surface/interface science into the era of molecular/atomic analysis. Atkin et al. [15] used atomic force microscopy (AFM) and tunneling scanning microscopy (TSM) to find a unique multilayer solvation layer at the interface between gold and ionic liquids. Han et al. [16] studied the electrocatalytic performance of platinum–carbon nanoparticles by means of modern electrochemical analysis. It was indicated that the electrocatalytic activity of platinum–carbon nanoparticles was closely related to their size and irregular surface. However, these surface/interface analysis techniques are mainly used to analyze and characterize the microscopic characteristics of fixed surfaces/interfaces. It is difficult to study the distribution and diffusion behavior of microscopic particles at liquid–liquid interfaces, such as oil–water interfaces.

Fortunately, molecular simulation provides another important means for exploring the distribution and movement of microparticles in the free interface region, and to some extent, makes up for the deficiency of modern surface/interface analysis technology. In recent years, molecular dynamics simulation has been used to study the distribution and movement of microscopic particles at the oil–water interface. Patel et al. [17] studied the structure orientation and hydrogen bonding of water molecules at the octane–water interface. The results indicated that the average number of hydrogen bonds and dielectric constant of water molecules at the interface were less than those in the water phase. Through the study of the hydrogen bond interaction between water molecules at the interface of dodecane phase and water phase, Chen et al. [18] pointed out that the slow hydrogen bond exchange between water molecules delayed the rearrangement of water molecules at the interface when the water phase and oil phase contact each other. Vatente et al. [19] used nonequilibrium molecular dynamics simulation to find that when potassium ions transfer from the water phase to the oil phase, it is necessary to strip the hydration layer molecules and overcome a certain energy barrier. Kadota et al. [20] pointed out that the mobility of ions at oil–water interface and the formation of organic molecular clusters could be effectively regulated by changing the types of organic molecules in the oil phase.

In conclusion, it has been confirmed that there are differences in the distribution and diffusion characteristics of microscopic particles between the oil–water interface and the oil–water phase, and some progress has been made in the molecular dynamics simulation of oil–water interfaces. However, the microscopic behaviors of particle distribution and diffusion in oil–water systems remain unclear. Especially, the mechanism behind the microscopic morphology of oil–water interfaces and the diffusion characteristics of microscopic particles near the interface when adding electrolyte ions in the aqueous phase are still to be explored. Therefore, molecular dynamics simulation is performed to study the distribution and diffusion behavior of microscopic particles at the interface of two phases in two oil–water systems of fluorobenzene–water and 1-pentanol–water, which are commonly used in pharmaceutical manufacturing. The effect of adding electrolyte ions (Na^+^/Cl^−^) to water on the interface morphology and water diffusion characteristics was analyzed in order to provide a clearer understanding of the distribution and diffusion of microscopic particles at the oil–water interface, which provides a theoretical basis for the design and control of physicochemical interface processes in engineering practice.

## 2. Molecular Dynamics Simulation

**Figure 1:**
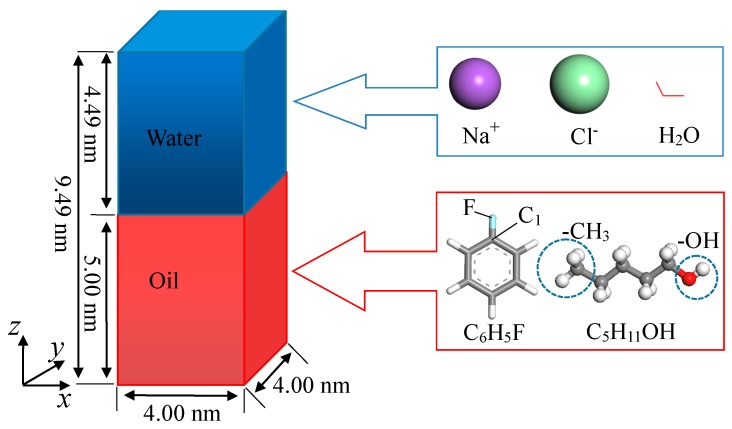
Model and molecular structure of the oil–water interface.

The structure of the oil–water system, the microstructure of water molecules, Na^+^/Cl^−^ ions, and oil phase molecules used in the molecular dynamics simulation study are depicted in Figure 1. As shown in the figure, the oil–water system consists of an upper water phase and a lower oil phase, forming an oil–water interface perpendicular to the *z* direction. The upper water phase contains 2000 water molecules (1.0 g/cm^3^), while the lower oil phase contains 300 fluorobenzene molecules (1.0 g/cm^3^) or 200 1-pentanol molecules (0.8 g/cm^3^). In the simulation, periodic boundary conditions are applied in the *x, y*, and *z* directions. The total thickness of the oil and water phases along the *z* direction is 9.49 nm, of which the oil phase thickness is about 5.00 nm, the water phase thickness is about 4.49 nm, and the area of the oil–water interface perpendicular to the *z* direction is 4.00 × 4.00 nm^2^. In this way, the oil and water phases in the *z* direction have enough thickness so that the interaction between them and the influence caused by the oil–water interface in the adjacent periodic cell can be neglected. Different quantities of electrolyte ions (Na^+^/Cl^−^) were added to the simulated water phase to investigate the impact of electrolyte ions on the distribution and diffusion characteristics of the microscopic particles at the oil–water interface. The number of molecules and ions in each simulation system is listed in Table 1. As is known, the mass concentration of saturated NaCl solution is 26.47% at room temperature. In our work, the simulation systems were all dilute solutions of NaCl (from 0.00% to 6.10%).

The interactions of microscopic particles decide the distribution and diffusion characteristics of particles at the oil–water interface; thus, the choice of molecular force field is quite significant in the calculation these properties. COMPASS (Condensed-phase Optimized Molecular Potentials for Atomistic Simulation Studies) force field [21] is an ab initio molecular force field, which can better deal with the interactions among inorganic and organic molecules and ions, and is suitable for the calculation of molecular structure and thermodynamic properties of condensed matter. Therefore, a COMPASS force field is used to calculate the interactions among water molecules, ions, and oil molecules. Bond length, bond angle, and dihedral angle are used to describe molecular structures. Lennard–Jones (LJ) potential and Coulomb potential are used to describe nonbonding interactions [22]. In order to eliminate unreasonable interaction between molecules and ions and optimize the initial particle distribution of the model system, steepest descent, conjugate gradient, and quasi-Newton methods were comprehensively used to optimize the initial configuration. On the basis of the initial configuration, we use canonical ensemble (NVT) to make the system balance via 1 ns of dynamic simulation, and then perform another 1 ns of molecular dynamics simulation. The simulation time step of which is 1 fs and the atomic coordinate information is saved every 10,000 steps. In the whole simulation process, the atom-based addition method is used to calculate Van der Waals forces, and the truncation radius is 1.2 nm. The Ewald summation method is applied in Coulomb force calculation [23] and the accuracy was 10^−3^ kcal/mol. A Nose heat bath [24] is adopted to control the temperature of the whole system during simulation at 298 K. All the sets and calculations stated above were conducted in the Materials Studio 6.0 packages [25].

**Figure 2:**
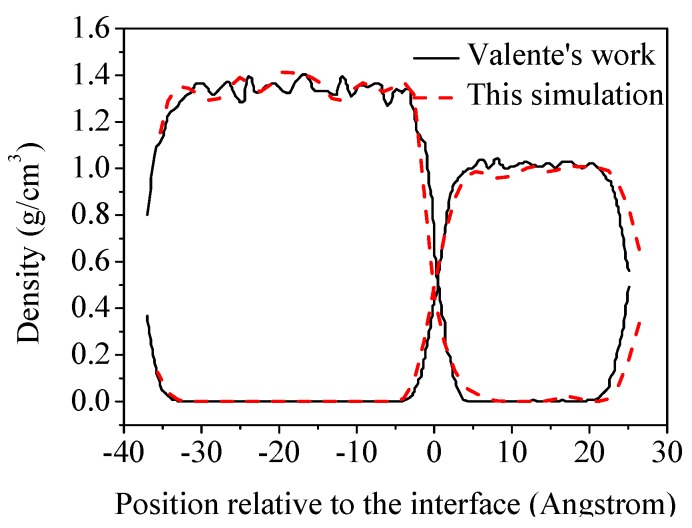
Comparison of concentration profiles of water and octane molecules at the oil–water interface between the present simulation and Valente’s work.

In order to verify the simulation method in our work, we simulate the molecular distributions of an octane–water system as provided by Valente [19]. The concentration distributions are compared in Figure 2. The agreement of the interfacial distribution profiles of water and octane molecules verifies the rationality of the method used in our work.

## 3. Results and Discussion

### 3.1. Microscopic Particle Distribution and Oil–Water Interface Morphology

**Figure 3:**
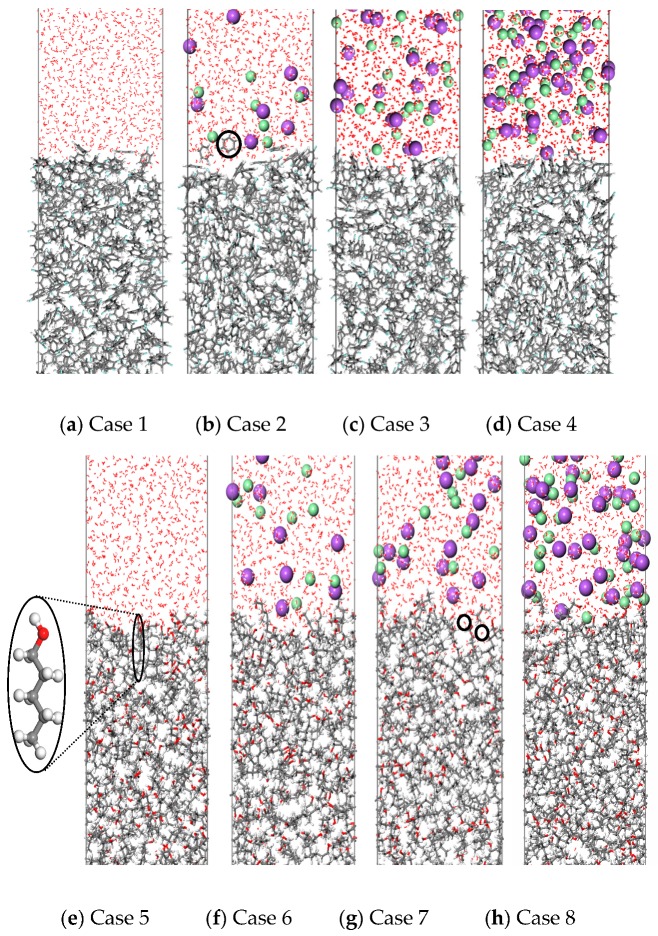
Simulation of oil–water interface in different systems after 1 ns molecular dynamics simulation: (**a**–**d**) Fluorobenzene–NaCl aqueous solution system containing different concentrations of ions; (**e**–**h**) 1-Pentanol–NaCl aqueous solution system containing different concentrations of ions.

In order to establish an intuitive understanding of the distribution characteristics of various microscopic particles at the oil–water interface, the interfacial microstructures of the fluorobenzene–water system and the pentanol–water system at equilibrium state with different electrolyte ion contents are presented in Figure 3. As shown in Figure 3a–d, fluorobenzene molecules are closely packed in the oil phase in the fluorobenzene–water system due to their planar configuration and small volume. Owing to the attraction of hydrogen bonds of adjacent water molecules, the arrangement of fluorobenzene molecules at the interface is looser than that of fluorobenzene molecules in the oil phase. When sodium ions and chloride ions exist in the aqueous phase, a small amount of fluorobenzene molecules enter the aqueous phase (as shown in Figure 3b) because of the additional ion–fluorobenzene electrostatic interaction. However, with the further increase of electrolyte ions (Na^+^/Cl^−^) in the aqueous phase, the density of aqueous phase layer increases, the electrostatic interaction between water molecules and anion and cation increases, thus the binding between water molecules and ions makes it difficult for fluorobenzene molecules to enter the aqueous phase (as shown in Figure 3d).

Compared with the fluorobenzene–water system, the pentanol molecule in the pentanol–water system has a larger volume and needs to overcome much larger steric hindrance in the process of movement and migration. Therefore, no pentanol molecule has been observed in the water phase, regardless of whether ions are added or not, as shown in Figure 3e–h. In addition, the amphiphilic nature of pentanol molecules makes the pentanol molecule at the interface point to the aqueous phase mainly with hydrophilic hydroxyl groups, as shown in Figure 3e. Moreover, owing to the large volume of pentanol molecules, loose accumulation, and large gaps between pentanol molecules at the interface, water molecules often enter the pentanol phase, as shown in Figure 3g.

**Figure 4:**
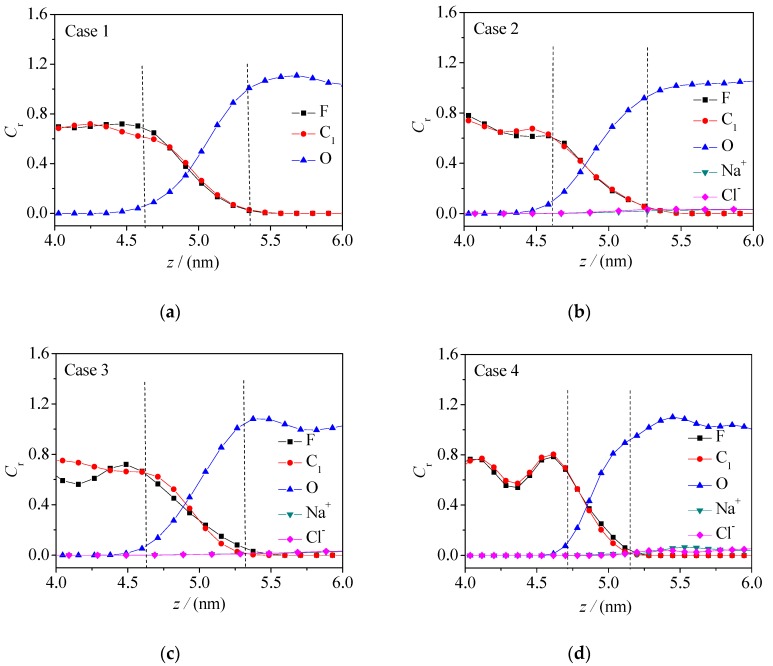
Concentration distribution curve of F, C, O, Na^+^, and Cl^−^ at the oil–water interface in the fluorobenzene–NaCl aqueous solution system: (**a**) Case 1; (**b**) Case 2; (**c**) Case 3; (**d**) Case 4.

**Figure 5:**
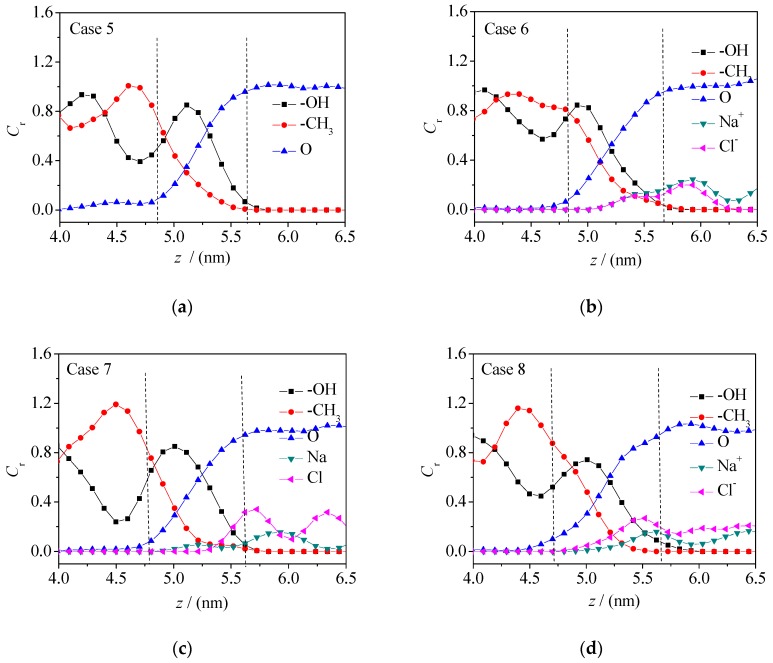
Concentration distribution curve of F, C, O, Na^+^, and Cl^−^ at the oil–water interface in the 1-pentanol–NaCl aqueous solution system: (**a**) Case 5; (**b**) Case 6; (**c**) Case 7; (**d**) Case 8.

Various particle concentration curves at the oil–water interface directly reflect the microscopic distribution characteristics of particles. The relative concentration distributions of various particles along the *z* direction is directedly shown in Figure 4 and Figure 5, respectively (*C*_r_, the ratio of the concentration of microscopic particles at the interface to the average concentration in the aqueous phase). The concentration curves of fluorine atoms (F) and α carbon atoms (C_1_) characterize the concentration distributions of fluorobenzene molecules and the concentration curves of hydroxyl (-OH) and methyl (-CH_3_) groups characterize the concentration distribution of pentanol molecules, while the concentration curve of oxygen atom (O) represents the concentration distribution of water molecules.

It can be seen from the figures that there is a interfacial zone between the oil and water phase (defined as the area where the concentration of oil phase molecule or water phase molecule is 10%–90% of their respective bulk phase concentration), that is, the oil–water interface region [26]. It can be seen that the oil–water interface thickness of the fluorobenzene–water system decreases with the increase of electrolyte ions (Na^+^/Cl^−^), while the oil–water interface thickness of the pentanol–water system increases with the increase of sodium ions and chloride ions, which is because the pentanol molecule has a longer alkyl chain than the fluorobenzene molecule, larger steric hindrance, and looser accumulation. In this way, when the ions in the water phase increase, the water molecules are squeezed, and the number of water molecules entering the interior of the pentanol phase increases, resulting in an increase of the volume of the oil–water interface. In addition, compared with the concentration curves of fluorine atoms (F) and α carbon atoms (C_1_), the concentration distribution curves of hydroxyl (-OH) and alkyl (-CH_3_) groups of pentanol molecules have concentration peaks near the oil–water interface, which are near the water and oil phases, respectively, indicating again that the amphiphilicity of pentanol molecule leads to an obvious orientation selectivity at the oil–water interface, that is, hydroxyl groups point towards the water phase and alkyl chains point towards the oil phase. Moreover, as can be seen from Figure 4 and Figure 5, the concentration curves of F and C_1_ at the interface basically coincide, which indicates that the fluorobenzene molecules at the interface do not show a specific orientation due to their small steric hindrance and easily achievable movement and rotation.

**Figure 6:**
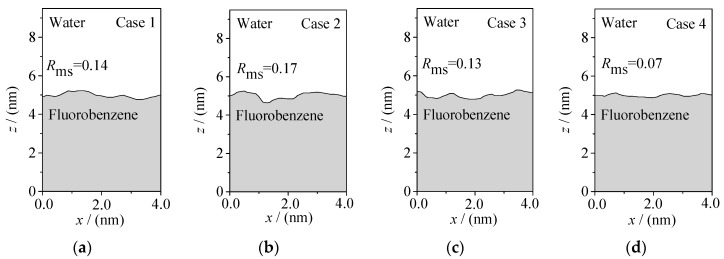
Interface morphology of the fluorobenzene–NaCl aqueous solution system with different contrations of ions: (**a**) Case 1; (**b**) Case 2; (**c**) Case 3; (**d**) Case 4.

**Figure 7:**
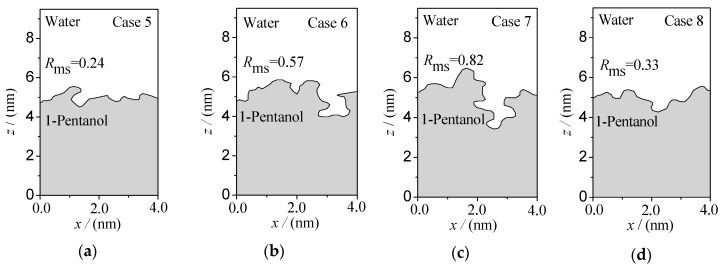
Interface morphology of the 1-pentanol–NaCl aqueous solution system with different concentrations of ions: (**a**) Case 5; (**b**) Case 6; (**c**) Case 7; (**d**) Case 8.

The rough morphology of the oil–water interface is also an important feature of the microscopic distribution of particles at the interface. Therefore, based on the spatial distribution of various particles in the oil–water two-phase systems, the oil–water interface boundary curves are generated by projecting the oil–water two-phase system to the x-y-z plane, as shown in Figure 6 and Figure 7. Here, the root mean square roughness *R*_ms_ [27] is especially defined to quantitatively characterize the roughness of an oil–water interface:

(1) $$R_{\mathrm{ms}}=\sqrt{\frac{1}{L}\overset{L}{\underset{0}{\int }}{\left[z\left(x\right)-\bar{z}\left(x\right)\right]}^{2}dx}$$

Among them, *L* is the length in the *x* direction and $z\left(x\right)-\bar{z}\left(x\right)$ is the difference between the *z* coordinates of each position on the interface boundary curve and the average of *z*. The *z* coordinates of different positions were obtained by Get Data software. Thus, for the fluorobenzene–water system (as shown in Figure 6), the roughness of the oil–water interface curve fluctuates slightly (*R*_ms_ = 0.07–0.17), and the root mean square roughness reaches the minimum (*R*_ms_ = 0.07) when the number of ions in the water phase reaches 40. It can be inferred that adding more ions in the water phase can reduce the roughness of the interface, which is due to the tight accumulation of molecules at the interface caused by the extrusion of water relative to the oil phase. Compared with the fluorobenzene–water system, the interfacial boundary curve of the pentanol–water system (as shown in Figure 7) fluctuates more obviously and its roughness is greater, which is mainly due to the large void of pentanol molecules, loose accumulation, and easy entry of water molecules into the void to form an irregular “erosion” morphology.

### 3.2. Diffusion Characteristics of Microscopic Particles at the Oil–water Interface

**Figure 8:**
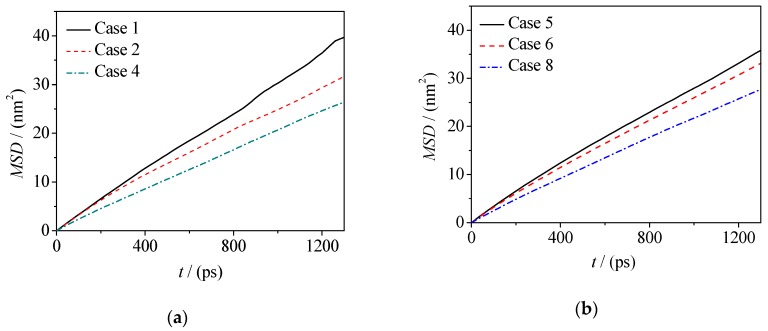
Mean displacement curve of water molecules in: (**a**) Fluorobenzene–NaCl aqueous solution system; (**b**) 1-Pentanol–NaCl aqueous solution system.

**Figure 9:**
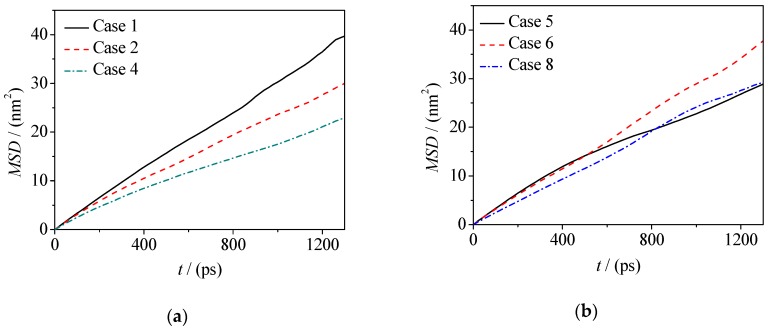
Mean displacement curve of water molecules at the interface: (**a**) Fluorobenzene–NaCl aqueous solution system; (**b**) 1-Pentanol–NaCl aqueous solution system.

Diffusion and migration of microscopic particles at oil–water interfaces are important for the distribution and rough morphology of those particles. The mean squared displacement (MSD) of microscopic particles can be used to characterize the difficulty of particle diffusion. The MSD (formula 2) of water molecules in the water phase and at the interface of two oil–water systems is calculated in this paper, and the MSD curves are plotted in Figure 8 and Figure 9.

(2) $$\mathrm{MSD}=\frac{1}{N}\overset{N}{\underset{i=1}{\sum }}\langle {\left|r_{i}\left(t\right)-r_{i}\left(0\right)\right|}^{2}\rangle$$

On this basis, the diffusion coefficient (*D*) of water molecules at the interface is calculated as following [28]:

(3) $$D=\frac{1}{6}\underset{t\to \infty }{\lim}\frac{dMSD}{dt}$$

In Formulas (2) and (3), the position coordinates of *r*_i_(0) and *r*_i_(*t*) are the position coordinates of the *i*-th water molecule at the initial time and the current time, respectively, and *D* is the diffusion coefficient of the water molecule. Accordingly, the corresponding diffusion coefficients are given in Table 2 (*D*_i_ is the diffusion coefficients of water molecules at the interface and *D*_b_ is the diffusion coefficients of water molecules in the water phase). From Formulas (2) and (3), it should be noted that for normal diffusion, the MSD-t plots should be linear and then the correct diffusion coefficient *D* can be obtained according to the hypothesis of Einstein function. Therefore, we extracted the data at the equilibrium stage.

By calculating the MSD curves and diffusion coefficients, it can be seen that adding electrolyte ions can reduce the diffusivity of water molecules in the fluorobenzene–water and pentanol–water systems (as shown in Figure 8, Figure 9 and Table 2), which is due to the fact that when electrolyte ions are added, the movement of water molecules not only overcomes the hydrogen bonding between them, but also overcomes the electrostatic interaction exerted by electrolyte ions. However, for water molecules at the oil–water interface, there are quite different situations in these two systems, that is, the diffusion ability of water molecules at the fluorobenzene–water interface decreases with the increase of the number of ions in the water phase, while the diffusion ability of water molecules at the pentanol–water interface increases first and then decreases with the increase of the number of ions in the water phase (as shown in Figure 8a,b and Table 2). In the fluorobenzene–water system, the extrusion of water molecules at the interface increases with the increase of the number of ions in the water phase. However, due to the tight accumulation of fluorobenzene molecules, this kind of squeezing action compresses the space of water molecules, resulting in a decrease in the diffusion capacity (as shown in Figure 3a–d and Figure 8). However, in the pentanol–water system, although the number of ions in the aqueous phase will still squeeze the water molecules at the interface, the water molecules can more easily enter the pentanol because of the loose accumulation of pentanol molecules at the interface. Therefore, this extrusion promotes the diffusion of water molecules into the voids of pentanol molecules, resulting in the enhancement of the diffusion ability of water molecules as the number of ions increases (as shown in Figure 3e–h and Figure 9). With the further increase of electrolyte ions in the aqueous phase, not only are the water molecules at the interface are squeezed, but so are the pentanol molecules. The gaps between pentanol molecules decrease, and the difficulty for water molecules to enter pentanol increases, resulting in a downward trend of diffusion ability of water molecules at the interface (Figure 9b).

**Figure 10:**
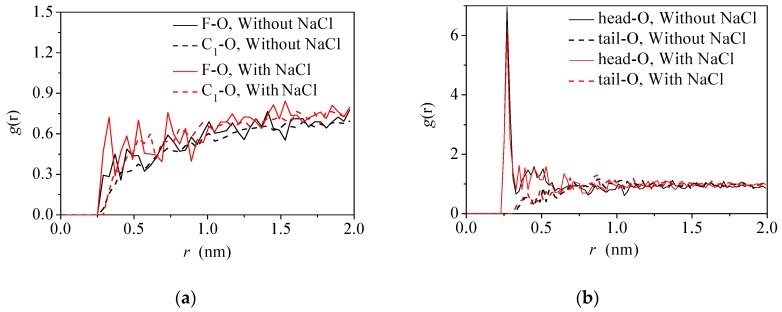
The radial distribution functions *g*(r) of hydrophilic and hydrophobic parts of organic solvents and water molecules at different oil–water interfaces: (**a**) The *g*(*r*) of C_1_-O and F-O in the fluorobenzene–water system; (**b**) The *g*(*r*) of head-O and tail-O in 1-the pentanol–water system.

In addition, the abnormal increase of the diffusion ability of water molecules at the pentanol–water interface with the increase of the number of ions in the water phase can be explained by radial distribution function (RDF) analysis. Figure 10a shows the *g*(*r*) of the hydrophilic and hydrophobic parts of fluorobenzene molecules around O (water) atoms for the fluorobenzene–water system with (without) Na^+^/Cl^−^ ions in the water phase. Larger peaks can be observed at ~0.3 nm in the *g*(*r*) of the hydrophilic groups and water molecules (F-O), especially for the system with Na^+^/Cl^−^ ions. This means the addition of NaCl leads to the compression of the interface and densely packing of the molecules. For the pentanol–water system (Figure 10b), more pronounced first peak was presented at the g(r) of hydrophilic part and water molecules (head-O). However, there is no obvious change in the *g*(*r*) profiles with and without NaCl in the water phase. It is suggested that the pentanol molecules at the interface orient their hydroxyl group toward the water phase. When adding NaCl in the water phase, some water molecules burst into the space of pentanol and the pentanol molecules retain regular configurations at the interface. Thus, the interfacial packing is not obviously changed.

## 4. Conclusions

In this paper, the distribution and diffusion behavior of microscopic particles at oil–water (fluorobenzene–water, 1-pentanol–water) two-phase interfaces were studied based on molecular dynamics simulation. The microscopic particle distribution, molecular orientation, interfacial roughness, and particle diffusion ability were analyzed. The effects of water molecular distribution and diffusion characteristics exerted on the types of organic molecules in the oil phase and the concentration of electrolyte ions (Na^+^/Cl^−^) in water on the oil–water interface were examined. The conclusions are as follows:

(1) The addition of a small amount of electrolyte ions in the water phase will promote the movement of fluorobenzene molecules towards the water phase at the oil–water interface, while the pentanol molecules at the oil–water interface cannot easily enter the water phase because of their large molecular size. The pentanol molecules show obvious orientation selectivity at the oil–water interface, i.e., hydroxyl groups orientate towards the water phase and alkyl chains orientate towards the oil phase. For water molecules, the close accumulation of fluorobenzene makes it difficult for water molecules to enter the fluorobenzene phase, whereas the larger voids between pentanol molecules facilitate the entry of water molecules.

(2) There is a microscopic particle interfacial zone at the oil–water interface. With the increase of the number of electrolyte ions, the water molecules at the interface are squeezed, and the accumulation of fluorobenzene is denser, which is not conducive to the entry of water molecules, and the thickness of the interfacial zone decreases. In contrast, the increase of the number of electrolyte ions promotes more water molecules to enter the pentanol phase with larger voids, which leads to the increase of the thickness of the interfacial zone.

(3) The root mean square roughness of oil–water interface increases first and then decreases with increasing of ion concentration in the water phase, and the interface roughness of the oil–water interface is larger than that of pentanol molecules.

(4) Whether in the fluorobenzene–water system or in the pentanol–water system, the diffusion coefficient of water molecules in the water phase decreases with the increase of the number of ions; the diffusion coefficient of water molecules in the fluorobenzene–water system decreases with the increase of the number of ions at the oil–water interface, while the diffusion coefficient of water molecules increases first and then decreases with the increase of the number of ions at the pentanol–water interface.

## Figures and Tables

**Table 1:** The number of microscopic particles contained in the various oil–water systems.

**Fluorobenzene–NaCl Aqueous Solution**	**Case Number**	**Case 1**	**Case 2**	**Case 3**	**Case 4**
	**Fluorobenzene**	300	300	300	300
	**Water Molecule**	2000	2000	2000	2000
	**Sodium Ion**	0	10	20	40
	**Chloride**	0	10	20	40
	**Mass Concentration**	------	1.60%	3.15%	6.10%
**1-Pentanol–NaCl Aqueous Solution**	**Case Number**	**Case 5**	**Case 6**	**Case 7**	**Case 8**
	**1-Pentanol**	200	200	200	200
	**Water Molecule**	2000	2000	2000	2000
	**Sodium Ion**	0	10	20	40
	**Chloride**	0	10	20	40
	**Mass Concentration**	------	1.60%	3.15%	6.10%

**Table 2:** Diffusion coefficients (in three-dimensional space) of water molecules in the water phase and at the interface with different oil–water interface systems (unit: 10^−10^ m^2^/s).

	**Diffusion Coefficient**	***D*_i_**	***D*_b_**
**Fluoro benzene–NaCl Aqueous Solution**	Case 1	0.45	0.50
	Case 2	0.37	0.39
	Case 4	0.28	0.32
	**Diffusion Coefficient**	***D*_i_**	***D*_b_**
**1-Pentanol–NaCl Aqueous Solution**	Case 5	0.33	0.43
	Case 6	0.47	0.40
	Case 8	0.38	0.35
